# The Efficacy of Topical Cefiderocol Treatment of Experimental Extensively Drug-Resistant *Pseudomonas aeruginosa* Keratitis Is Dependent upon the State of the Corneal Epithelium

**DOI:** 10.3390/antibiotics13100979

**Published:** 2024-10-17

**Authors:** Eric G. Romanowski, Jonathan B. Mandell, Vishal Jhanji, Robert M.Q. Shanks

**Affiliations:** The Charles T. Campbell Ophthalmic Microbiology Laboratory, UPMC Vision Institute, Department of Ophthalmology, University of Pittsburgh School of Medicine, Pittsburgh, PA 15219, USA; jbm42@pitt.edu (J.B.M.); jhanjiv@pitt.edu (V.J.); shanksrm@upmc.edu (R.M.Q.S.)

**Keywords:** *Pseudomonas aeruginosa*, keratitis, corneal epithelium, antibiotic penetration, extensively drug-resistant, cefiderocol, tobramycin, ciprofloxacin, animal model

## Abstract

**Background:** An overlooked factor in the efficacy of topical antibiotics to treat bacterial keratitis is the state of the corneal epithelium. Recently, we evaluated topical cefiderocol for the treatment of extensively drug-resistant (XDR) *Pseudomonas aeruginosa* (PA) keratitis in eyes with the corneal epithelium abraded. The goal of this study was to use the same model with the corneal epithelium left intact to evaluate the efficacy of cefiderocol and other antibiotics and compare the results to those of the previous study. **Methods:** NZW rabbit corneas with intact epithelium were inoculated with XDRPA. After 16 h, the rabbits were topically treated with cefiderocol 50 mg/mL, ciprofloxacin 0.3%, tobramycin 14 mg/mL, or saline. Following 8 h of treatment, the corneas were harvested for CFU determinations and cefiderocol concentrations. **Results:** Only cefiderocol significantly decreased CFU of the XDRPA strain compared with saline. The CFU in the cefiderocol and tobramycin-treated corneas for the XDRPA strain with initially intact epithelium were 1.83–1.4 Log_10_ greater than those produced in corneas with the abraded epithelium (*p* < 0.05). Cefiderocol concentrations were 5.02× less in corneas with initially intact epithelium. **Conclusions:** The efficacy of cefiderocol and tobramycin to treat experimental XDRPA keratitis is dependent on the state of the corneal epithelium.

## 1. Introduction

The successful topical treatment of corneal infections depends upon a number of factors. Most importantly, the infecting bacteria and its antibiotic susceptibility profile necessitate the choice of the appropriate antibiotic. Other factors that affect the efficacy of topical antibiotics include lipid and water solubilities, polarity, and their ophthalmic formulations (pH, concentration of the antibiotic, and additives), which can promote high corneal antibiotic concentrations [1]. For example, the addition of the preservative benzalkonium chloride to 0.3% gatifloxacin increased its efficacy in the topical treatment of an experimental *Staphylococcus aureus* corneal infection in a NZW rabbit model, presumably through the toxic effects of benzalkonium chloride on the corneal epithelium, resulting in increased corneal concentrations of gatifloxacin [2]. Microdisruption of the corneal epithelium through the “microtoxicity” of preservatives is thought to enhance drug penetration through the corneal epithelium, which is the principal barrier to drug penetration [3,4].

Most bacterial corneal infections present to ophthalmologists as ulcers in which the corneal epithelium has eroded. An overlooked factor in the efficacy of topical antibiotics to treat bacterial keratitis is the size of the corneal epithelial defect. It is known that topical antimicrobials reach higher concentrations in the cornea and aqueous humor when the epithelium is removed [5,6,7,8,9,10]. Higher corneal concentrations of antibiotics are expected to result in increased efficacy, reaching bactericidal and mutant prevention concentrations.

Therefore, the effectiveness of topical antibiotics should be evaluated in experimental models to determine whether higher corneal concentrations, in eyes with the corneal epithelium removed, translate into increased efficacy. We have previously shown that removing the corneal epithelium from rabbit eyes increased the antibacterial efficacy of topical 5% and 2.5% concentrations of the glycopeptide vancomycin, but not 1.25% vancomycin in an MRSA rabbit keratitis model [11]. In addition, the removal of the corneal epithelium significantly increased the topical antibacterial efficacy of an antimicrobial peptide mimetic, Brilacidin, in the same MRSA rabbit keratitis model [12]. Furthermore, the corneal epithelium was shown to act as a barrier that resulted in decreased topical antibacterial efficacy of the beta-lactam penicillin [13] also in that same model. In contrast, Kupferman and Leibowitz showed that the corneal epithelium did not affect the antibacterial efficacy of several topical antibiotic formulations (chlortetracycline 1.0% ointment, erythromycin 0.5% ointment, gentamicin 0.3% solution and ointment, neomycin 0.5% solution, and tetracycline 1.0% ointment) in a *Staphylococcus aureus* rabbit keratitis model [14]. These contrasting results suggest that the ability of a topical antimicrobial to penetrate the corneal epithelium is antimicrobial-dependent.

Recently, we published a study evaluating two standard-of-care antibiotics for Gram-negative keratitis (tobramycin 14 mg/mL and ciprofloxacin 0.3%) and a potentially new antibiotic (cefiderocol [15,16,17,18] 50 mg/mL) for the treatment of extensively drug-resistant (XDR) *Pseudomonas aeruginosa* (PA) keratitis [19]. This study was in response to an outbreak of XDRPA keratitis traced to contaminated multiuse bottles of artificial tears purchased online [20,21,22,23,24,25]. The outbreak was caused by a Sequence Type 1203 PA strain and associated with severe corneal infections [22,23,24,25]. We demonstrated that topical cefiderocol 50 mg/mL was effective in reducing XDRPA colony counts in infected rabbit corneas when the epithelium was removed to mimic severe corneal ulceration [19]. Cefiderocol was more effective than both tobramycin and ciprofloxacin, the latter of which had no effect [19].

Based on these data alone, we cannot determine the ability of cefiderocol, tobramycin, and ciprofloxacin to penetrate the corneal epithelium when there are small or no corneal epithelial defects, as compared to large defects. These antibiotics represent three distinct classes of antibiotics (siderophore cephalosporin, aminoglycoside, and fluoroquinolone, respectively) whose ability to penetrate the corneal epithelium is unknown. This led us to the current study for which the goal was to determine the antibacterial efficacy of topical cefiderocol, tobramycin, and ciprofloxacin in the treatment of antibiotic-susceptible PA and XDRPA infections in eyes with intact corneal epithelium at the time of inoculation. We compared these data to the data from eyes with the corneal epithelium abraded at the time of inoculation, which was generated in our previous study [19]. Historical data were used to reduce the number of animals needed for this study. These combined studies will provide insight into the penetration and antibacterial efficacy of these antibiotics from distinct antibiotic classes in PA-infected corneas depending on the state of the corneal epithelium.

## 2. Results

### 2.1. State of the Corneal Epithelium

**Figure 1:**
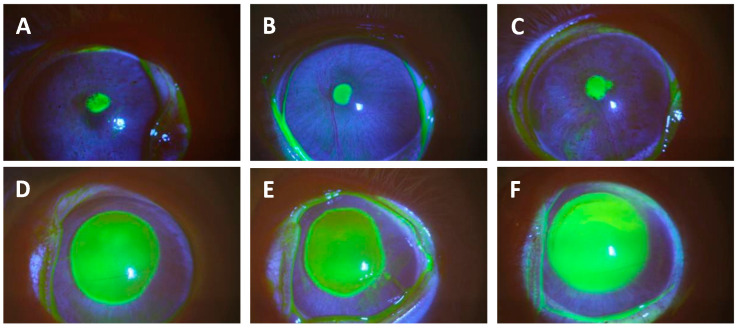
This figure presents photographs of corneas stained with 1% sodium fluorescein from eyes infected with XDRPA CDC1270 after 16 h of infection. Yellow staining shows epithelial defects. Photographs (**A**–**C**) are small defects from eyes that initially had intact epithelium, but ulcers had formed during the infection period. These were representative of the small ulcers described in this study. Photographs (**D**–**F**) show large defects from eyes that had their epithelium abraded with an Amoils Epithelial Scrubber prior to inoculation. These images were from animals used in a prior study [19] but have not been presented in a publication.

Initially, the corneal epithelium of the eyes of the rabbits in the current study was intact at the time of inoculation. We did not examine the eyes prior to the onset of therapy for the K900 (an antibiotic-susceptible *P. aeruginosa* strain isolated from a patient with a corneal ulcer)-infected animals and in the first trial of rabbits infected with the CDC1270 (XDRPA strain isolated from the cornea of a patient from the 2023 artificial tears infection outbreak) isolate. Subsequently, eyes infected with CDC1270 from the Onset of Therapy group were examined using a slit lamp and stained with 1% sodium fluorescein prior to euthanasia to determine whether any epithelial defects were produced over the 16 h infection in the final trial. Fluorescein staining revealed that 1–2 mm epithelial defects were produced over the 16 h infection period with a mean pixel area of 73,176 ± 9594 pixels (ImageJ). These defects were significantly smaller than the 6 mm defects induced in our previous study [19], which increased in size to 7–8 mm over the 16 h infection and had a mean area of 1,170,905 ± 206,856 pixels (*p* = 0.012; *t*-test) (Figure 1). As the defects were not measured in the eyes infected with K900, we assume that there were similar small or no epithelial defects present at the onset of therapy. 

### 2.2. Antimicrobial Efficacy in the NZW Rabbit Corneas Infected with P. aeruginosa

Table 1 presents the Log_10_ mean ± standard deviation corneal CFU for each topical treatment and bacterial strain in rabbit corneas intact epithelium at the time of inoculation (Intact). Also included in Table 1 are the data from our companion study with the corneal epithelium abraded at the time of inoculation resulting in the large 7–8 mm epithelial defects that mimicked a severe corneal ulcer (Abraded) [19].

For corneas infected with the CDC1270 isolate, only cefiderocol significantly reduced CDC1270 CFU compared to the saline control in eyes with initially intact corneal epithelium. Cefiderocol produced a 2-Log_10_ (99%) decrease in CDC1270 corneal colony counts after 8 h of treatment, whereas tobramycin and ciprofloxacin did not produce significant decreases (>1 Log_10_) in CFU compared to the saline control in eyes with initially intact corneal epithelium (first data column of Table 1). This is unsurprising because the CDC1270 isolate is highly resistant to ciprofloxacin and tobramycin. In addition, cefiderocol significantly decreased the CFU compared to ciprofloxacin in eyes with initially intact corneal epithelium, but not compared to tobramycin. Although there may be small differences and overlapping standard deviations in the colony count data among the treatment groups, the statistical analysis nevertheless demonstrates that cefiderocol significantly decreased the number of CDC1270 CFU in the cornea after only 8 h of treatment in eyes in which the corneal epithelium was initially intact. These data suggest that cefiderocol can penetrate through a non-abraded corneal epithelium in high enough concentrations to be effective in the treatment of an XDR *P. aeruginosa* corneal infection in the corneal stroma that is susceptible to cefiderocol.

Comparing the CDC1270 CFU in corneas with initially intact corneal epithelium to the data from corneas with abraded corneal epithelium from our previous study [19], corneas treated with cefiderocol and tobramycin demonstrated significantly lower colony counts in corneas with abraded corneal epithelium compared to corneas with initially intact corneal epithelium. For cefiderocol, the CFU reduction was 1.83-Log_10_ greater in the abraded corneal epithelium vs. initially intact corneal epithelium. A significant reduction in CFU was also observed with tobramycin (1.4-Log_10_) for the CDC1270 strain (*p* < 0.001). There was a trend towards lower CFU for the ciprofloxacin-treated eyes with the abraded corneal epithelium [19] compared with the eyes with initially intact corneal epithelium seen in this study for the CDC1270 strain (0.9-Log_10_, *p* = 0.10) (row comparisons in Table 1). There was no significant difference in corneal CFU at the onset of therapy for CDC1270-infected eyes with initially intact or abraded epithelium (row comparison in Columns 1 and 2).

In contrast to the CDC1270 strain, all antibiotics significantly reduced the CFU of the antibiotic-susceptible K900 strain compared to its saline control in corneas with initially intact corneal epithelium (data column 3 in Table 1). A similar result was demonstrated in corneas with abraded corneal epithelium from our previous study (data column 4 in Table 1) [19]. There were no significant differences for any of the antibiotics or the saline and Onset of Therapy controls when comparing the CFU from corneas with initially intact corneal epithelium or abraded corneal epithelium (row comparisons from columns 3 and 4 in Table 1). There were no significant differences in CFU between eyes with initially intact corneal epithelium and eyes with abraded corneal epithelium for the K900 strain (row comparisons in Table 1). This is due to the low MICs of the K900 strain to cefiderocol, tobramycin, and ciprofloxacin since lower corneal concentrations are necessary to successfully treat corneal infections.

### 2.3. Approximate Corneal Penetration and Concentrations of Cefiderocol

After CFU determination, the homogenates of the cefiderocol-treated corneas were filter sterilized, and the approximate cefiderocol concentrations from each cornea were determined as described previously [19]. These data were separated for the bacterial isolates. It must be noted that three of the homogenates from the corneas with initially intact corneal epithelium in the K900-infected corneas and one cornea from the CDC1270-infected corneas produced cefiderocol concentrations less than the limit of detection of the assay. Therefore, those samples were considered to have 0 µg/mL concentrations of cefiderocol for statistical purposes but are likely to have cefiderocol concentrations in their corneas. These data are presented in Table 2.

The mean corneal cefiderocol concentrations for corneas with initially intact corneal epithelium (intact) were significantly lower than those with abraded corneal epithelium (abraded) for both bacterial groups. For eyes infected with K900 with initially intact corneal epithelium, the cefiderocol concentration was 21.5-fold less compared to eyes with abraded corneal epithelium. However, for the CDC1270-infected eyes, the difference was only 5.02-fold. There were no significant differences between the cefiderocol concentrations between the K900- and CDC1270-infected corneas for initially intact corneal epithelium (*p* = 0.223) and abraded corneal epithelium (*p* = 0.781, *t*-Test).

## 3. Discussion

It has been previously shown that topical antimicrobials reach higher concentrations in the cornea and aqueous humor when the epithelium is removed [3,4]. We have also demonstrated an increase in efficacy of a potential ocular antimicrobial Brilacidin [12], several concentrations of the glycopeptide, vancomycin [11], and the beta-lactam, penicillin [13] in corneas with the epithelium removed compared to corneas that had intact epithelium. While this has been demonstrated for an antimicrobial peptide, a glycopeptide, and a beta-lactam, it has not been shown for other antibiotic classes. It is also imperative to explore this issue with a new antibiotic, like cefiderocol, which could be used clinically to treat serious keratitis caused by extremely drug-resistant bacteria seen in the recent outbreak associated with artificial tears [20,21,22,23,24,25].

The results of the current study demonstrate that the corneal epithelium can act as a barrier for the penetration and subsequent antimicrobial efficacy of the siderophore cephalosporin, cefiderocol, and the aminoglycoside tobramycin, in the treatment of an experimental XDRPA infection. Significantly lower bacterial loads of XDRPA were produced in the corneas of the cefiderocol- and tobramycin-treated groups from our previous study that had 6 mm areas of the epithelium mechanically abraded [19] compared to the antibiotic-treated corneas in which the epithelium was left intact at the time of inoculation in the current study.

The intent of the current study was to have the corneal epithelium intact to determine whether it acted as a barrier to drug penetration. As such, the corneas were not examined for corneal defects at the onset of therapy 16 h post-infection. In the final trial with the CDC1270 strain, the corneas were stained with sodium fluorescein. At this time, small 1–2 mm diameter corneal defects were discovered following the 16 h infection period prior to the onset of therapy in these non-abraded corneas. As such, we do not know whether an epithelial defect formed during the 16 h infection period for the K900-infected eyes. Therefore, we assume there was a small corneal defect or no defect at all in the K900-infected eyes. The data from this study demonstrated that there was a 21.5-fold lower cefiderocol concentrations in the non-abraded corneas, compared to the abraded corneas with large corneal epithelial defects. In addition, although not statistically significant, the cefiderocol concentration in the K900-infected corneas was 4.55-fold lower than the cefiderocol concentrations in the CDC1270-infected corneas initially intact corneal epithelium compared to a 1.06-fold decrease in corneas with abraded corneal epithelium. This suggests there was a largely or completely intact corneal epithelium in the K900-infected corneas that further reduced the penetration of cefiderocol into those corneas.

For the CDC1270-infected eyes, it was noted in the previous study that the defects in the epithelium of the abraded corneas similarly increased by 1–2 mm in diameter over the 16 h infection period prior to treatment. These data were not presented in the original study.

For the XDRPA-infected eyes treated with cefiderocol, the antibacterial efficacy in eyes with initially intact corneal epithelium was substantially lower than those with abraded corneal epithelium. This outcome is consistent with the concentration of cefiderocol being 5.02× greater in eyes with an abraded corneal epithelium [19] compared to those with initially intact corneal epithelium. We did not measure the corneal concentrations produced by tobramycin 14 mg/mL and ciprofloxacin 0.3% but, presumably, they were also higher in the abraded corneas.

While we did see a significant increase in the effectiveness of cefiderocol and tobramycin when the corneal epithelium was abraded, this does not diminish the findings that all the test antibiotics demonstrated significant reductions in bacterial colony counts in the corneas with initially intact corneal epithelium compared to the saline control for the antibiotic-susceptible PA strain K900. This demonstrates that the antibiotics can penetrate the corneal epithelium in concentrations high enough to effectively treat antibiotic-susceptible PA infections.

Although we did not see a significant decrease in CFU for ciprofloxacin-treated eyes with abraded corneal epithelium compared to those with initially intact corneal epithelium for the CDC1270 strain, there was a 0.9 Log_10_ reduction in CFU, suggesting that the corneal epithelium may inhibit the achievement of very high corneal concentrations of ciprofloxacin needed in the cornea to successfully reduce corneal CFU of the XDRPA CDC1270 strain. Published images of patient eyes infected with the XDRPA strain from the recent outbreak demonstrated large corneal ulcers [20,26]. We expect that eyes infected with this strain will generally develop large ulcers, which would favor antibiotic penetration.

We also saw a trend toward a significant increase in corneal colony counts for the antibiotic-susceptible K900 eyes with initially intact corneal epithelium treated with cefiderocol compared to the eyes with abraded corneal epithelium. This further illustrates that although topical cefiderocol is effective, the state of the corneal epithelium can play a role in its efficacy.

Most importantly, our study demonstrates that topical cefiderocol was the only test antibiotic to significantly reduce corneal colony counts of an XDRPA strain, which is resistant to all other antibiotics. Cefiderocol was effective regardless of the state of the corneal epithelium, although it was more effective when the epithelium was removed. It is noteworthy though that PA infections are usually associated with medium to large epithelial defects [27]. The data from this study and the previous study demonstrate that cefiderocol can be an effective last line of defense for the treatment of severe PA infections resistant to the standard-of-care antibiotic treatments for PA keratitis.

## 4. Materials and Methods

### 4.1. Bacterial Isolates

The XDRPA strain CDC1270 (AR-1270) was isolated from the cornea of a patient from the 2023 artificial tears infection outbreak [19] and was obtained from the Centers for Disease Control and Prevention (CDC). The Minimum Inhibitory Concentrations (MIC) of the CDC1270 isolate to the antibiotics used in this study are cefiderocol 0.125 µg/mL (susceptible); ciprofloxacin > 32 µg/mL (resistant); and tobramycin > 256 µg/mL (resistant) [15].

The deidentified antibiotic-susceptible K900 *P. aeruginosa* strain was isolated from a patient with a corneal ulcer that presented to the Charles T. Campbell Ophthalmic Microbiology Laboratory at the Department of Ophthalmology at the University of Pittsburgh School of Medicine. The MICs for K900 are cefiderocol 0.023 µg/mL (susceptible); ciprofloxacin 0.19 µg/mL (susceptible); and tobramycin 0.5 µg/mL (susceptible) [19]. Susceptibility is based on the systemic CLSI breakpoints. There are no susceptibility standards for topical ocular therapy.

### 4.2. Topical Agents

All topical agents used were purchased from the inpatient hospital pharmacy at UPMC Presbyterian. Cefiderocol 50 mg/mL was prepared from IV FEDROJA (cefiderocol for injection, Shionogi Inc., Florham Park, NJ, USA) and was stored in a refrigerator and out of light, as previously described [19]. The cefiderocol was freshly prepared for each in vivo trial. Fortified tobramycin (TOBRAMYCIN INJECTION USP, 40 mg/mL, Fresenius Kabi USA, Lake Zurich, IL, USA) was also freshly prepared for each rabbit trial as described [19]. A commercial ophthalmic formulation of ciprofloxacin 0.3% (ciprofloxacin hydrochloride ophthalmic solution, 0.3%, Sandoz Inc., Princeton, NJ, USA) was used in this study. Sodium chloride injection USP 0.9% (saline) (Baxter Healthcare Corp., Deerfield, IL, USA) served as the negative control and as the diluent for the cefiderocol and tobramycin.

### 4.3. Animals

New Zealand White (NZW) specific pathogen-free female rabbits (1.1–1.4 kg) were purchased from Charles River Laboratories’ Canadian rabbitry. The rabbit experiments adhered to the ARVO Statement on the Use of Animals in Ophthalmic and Vision Research. Approval was obtained from the University of Pittsburgh’s IACUC prior to the start of the study (Protocol #23053154).

### 4.4. Experimental Protocol

Duplicate trials containing 15 NZW rabbits each (a total of 30 rabbits) for each of the two *P. aeruginosa* strains (K900 and CDC1270) were conducted. On the days of the experiments, rabbits received intramuscular injections of 40 mg/kg of ketamine (Ketamine Hydrochloride Injection 100 mg/mL, Dechra Veterinary Products, Overland Park, KS, USA) and 4 mg/kg of xylazine (Xylazine Injection 100 mg/mL, Covetrus North America, Dublin, OH, USA) to induce systemic anesthesia. Topical 0.5% proparacaine (Proparacaine Hydrochloride Ophthalmic Solution, USP 0.5%, Bausch & Lomb, Tampa, FL, USA) was used to anesthetize the corneas. The rabbits were placed on a portable operating table under a surgical microscope. The eyes were proptosed using a dacron-tipped applicator and the cornea of one eye from each rabbit was intrastromally injected with approximately 2300 colony-forming units (CFU) of the *P. aeruginosa* strains in 25 µL of phosphate-buffered saline (PBS). The rabbits were allowed to recover from anesthesia and placed in their housing rooms.

Sixteen hours post-inoculation, the rabbits were divided into 5 groups of 3 rabbits per trial for a total of 6 rabbits per group. The groups were as follows: (A) cefiderocol 50 mg/mL; (B) ciprofloxacin 0.3% (Standard of Therapy Control); (C) tobramycin 14 mg/mL (Standard of Therapy Control); (D) saline (negative control); and (E) No Treatment (Onset of Therapy Control). The Onset of Therapy Control contained rabbits euthanized at this time to provide the baseline bacterial counts in the corneas at the onset of topical treatment. The rabbits were euthanized with overdose intravenous injections of Euthasol solution (EUTHASOL^®^ [pentobarbital sodium and phenytoin sodium] Euthanasia Solution, Virbac Corporation, Kansas City, MO, USA) following systemic anesthesia with ketamine and xylazine in accordance with the 2020 AVMA Guidelines for Euthanasia of Animals. The corneas were then harvested and processed for bacterial load, as previously described [19].

Topical treatments for all groups were started at this time. The treatment regimen was 1 drop every 15 min for 1 h, and then every 30 min for 7 h (19 total drops over 8 h).

The corneas from one Onset of Therapy group for rabbits inoculated with CDC1270 were examined with a slit lamp and stained with fluorescein (AK-FJUOR^®^ 10% Fluorescein Injection USP, Akorn, Inc., Lake Forest, IL, USA) prior to euthanasia to determine if there was a corneal ulcer present. The eyes were photographed at that time and the diameter of the epithelial defects was measured using a standard ruler.

Following all topical treatments, the rabbits were euthanized and the corneas were harvested and processed as described above.

The approximate cefiderocol corneal concentrations were determined using a bioassay and a Linear Fitted Line Plot Regression Analysis, as described previously [19]. Corneal concentrations were not determined for tobramycin and ciprofloxacin.

### 4.5. Statistical Analysis of Corneal Colony Counts

The corneal colony counts + 1 were Log_10_ transformed and analyzed using ANOVA with Tukey’s multiple comparisons test (GraphPad Prism Version 9.5, Boston, MA, USA) to determine the effectiveness of the antibiotics. The comparison of corneal concentrations between corneas with initially intact corneal epithelium and abraded corneal epithelium and the areas of the epithelial defects determined using ImageJ Version 1.54 (National Institutes of Health, Bethesda, MD, USA) were analyzed using a Two-sample *t*-Test (Minitab Version 19, State College, PA, USA).

## Figures and Tables

**Table 1:** Mean ± SD Log_10_ corneal *P. aeruginosa* colony counts (*n* = 6 per group).

	CDC1270			K900		
	CDC1270		Intact vs. Abraded	K900		Intact vs. Abraded
	Intact	Abraded [19]	*p* Value	Intact	Abraded [19]	*p* Value
Cefiderocol	3.09 ± 0.83 ^#$^	1.26 ± 1.43 ^#$&^	*p* = 0.02	1.85 ± 1.47 ^#^	0.53 ± 0.83 ^#^	*p* = 0.08 NS
Ciprofloxacin	4.43 ± 1.12	3.53 ± 0.38	*p* = 0.10 NS	0.88 ± 0.97 ^#^	0.56 ± 0.91 ^#^	*p* = 0.57 NS
Tobramycin	4.09 ± 0.62	2.69 ± 0.40 ^#^	*p* < 0.001	0.75 ± 0.83 ^#^	0.77 ± 0.85 ^#^	*p* = 0.97 NS
Saline	5.09 ± 0.32	4.62 ± 0.80	*p* = 0.21 NS	3.65 ± 0.30	2.93 ± 1.00	*p* = 0.12 NS
Onset of Therapy	4.46 ± 0.95	3.74 ± 1.02	*p* = 0.23 NS	3.92 ± 0.54	3.87 ± 0.85	*p* = 0.93 NS

^#^ *p* < 0.05 vs. comparable saline control in column (ANOVA); ^$^ *p* < 0.05 vs. comparable ciprofloxacin group in column (ANOVA); ^&^ *p* < 0.05 vs. comparable tobramycin group in column (ANOVA). NS = Not Significant.

**Table 2:** Mean ± SD approximate corneal cefiderocol concentrations [µg/mL] (*n* = 6 per group).

	Intact	Abraded	*p* Value	Fold Difference
K900-Infected Eyes	1.08 ± 1.18	23.13 ± 8.80	*p* = 0.002	21.5
CDC1270-Infected Eyes	4.91 ± 6.64	24.63 ± 9.34	*p* = 0.002	5.02

## Data Availability

The data reported in this study are available in this manuscript.

## References

[1] Benson H. (1974). Permeability of the cornea to topically applied drugs. Arch. Ophthalmol..

[2] Romanowski E.G., Mah F.S., Kowalski R.P., Yates K.A., Gordon Y.J. (2008). Benzalkonium chloride enhances the antibacterial efficacy of gatifloxacin in an experimental rabbit model of intrastromal keratitis. J. Ocul. Pharmacol. Ther..

[3] Snyder R.W., Glasser D.B. (1994). Antibiotic therapy for ocular infection. West. J. Med..

[4] López Bernal D., Ubels J.L. (1991). Quantitative evaluation of the corneal epithelial barrier: Effect of artificial tears and preservatives. Curr. Eye Res..

[5] Fukuda M., Inoue A., Sasaki K., Takahashi N. (2004). The effect of the corneal epithelium on the intraocular penetration of fluoroquinolone ophthalmic solution. Jpn. J. Ophthalmol..

[6] O’Day D.M., Head W.S., Robinson R.D., Clanton J.A. (1986). Corneal penetration of topical amphotericin B and natamycin. Curr. Eye Res..

[7] Sakarya R., Sakarya Y., Ozcimen M., Kesli R., Alpfidan I., Kara S. (2013). Ocular penetration of topically applied 1% daptomycin in a rabbit model. J. Ocul. Pharmacol. Ther..

[8] Wei L.C., Tsai T.C., Tsai H.Y., Wang C.Y., Shen Y.C. (2010). Comparison of voriconazole concentration in the aqueous humor and vitreous between non-scraped and scraped corneal epithelium groups after topical 1% voriconazole application. Curr. Eye Res..

[9] Zhang J., Wang L., Zhou J., Zhang L., Xia H., Zhou T., Zhang H. (2014). Ocular penetration and pharmacokinetics of topical clarithromycin eye drops to rabbits. J. Ocul. Pharmacol. Ther..

[10] Bron A.M., Péchinot A., Garcher C., Guyonnet G., Kazmierczak A. (1992). Ocular penetration of topically applied norfloxacin 0.3% in the rabbits and in humans. J. Ocul. Pharmacol..

[11] Romanowski E.G., Romanowski J.E., Shanks R.M.Q., Yates K.A., Mammen A., Dhaliwal D.K., Jhanji V., Kowalski R.P. (2020). Topical vancomycin 5% is more efficacious than 2.5% and 1.25% for reducing viable MRSA in infectious keratitis. Cornea.

[12] Kowalski R.P., Romanowski E.G., Yates K.A., Mah F.S. (2016). An independent evaluation of a novel peptide mimetic, Brilacidin (PMX30063), for ocular anti-infective therapy. J. Ocul. Pharmacol. Ther..

[13] Kowalski R.P., Romanowski E.G., Yates K.A., Romanowski J.E., Grewal A., Bilonick R.A. (2018). Is there a role for topical penicillin treatment of *Staphylococcus aureus* keratitis based on elevated corneal concentrations?. J. Clin. Ophthalmol. Optom..

[14] Kupferman A., Leibowitz H.M. (1977). Topical antibiotic therapy of staphylococcal keratitis. Arch. Ophthalmol..

[15] Zhanel G.G., Golden A.R., Zelenitsky S., Wiebe K., Lawrence C.K., Adam H.J., Idowu T., Domalaon R., Schweizer F., Zhanel M.A. (2019). Cefiderocol: A siderophore cephalosporin with activity against carbapenem-resistant and multidrug-resistant gram-negative bacilli. Drugs.

[16] El-Lababidi R.M., Rizk J.G. (2020). Cefiderocol: A siderophore cephalosporin. Ann. Pharmacother..

[17] McCreary E.K., Heil E.L., Tamma P.D. (2021). New perspectives on antimicrobial agents: Cefiderocol. Antimicrob. Agents Chemother..

[18] Yao J., Wang J., Chen M., Cai Y. (2021). Cefiderocol: An overview of its in-vitro and in-vivo activity and underlying resistant mechanisms. Front. Med..

[19] Romanowski E.G., Mumper S.M., Shanks H.Q., Yates K.A., Mandel J.B., Zegans M.E., Shanks R.M.Q. (2024). Cefiderocol is an effective topical monotherapy for experimental extensively-drug resistant *Pseudomonas aeruginosa* keratitis. Ophthalmol. Sci..

[20] Morelli M.K., Kloosterboer A., Fulton S.A., Furin J., Newman N., Omar A.F., Rojas L.J., Marshall S.H., Yasmin M., Bonomo R.A. (2023). Investigating and Treating a Corneal Ulcer Due to Extensively Drug-Resistant *Pseudomonas aeruginosa*. Antimicrob. Agents Chemother..

[21] Shoji M.K., Gutkind N.E., Meyer B.I., Yusuf R., Sengillo J.D., Amescua G., Miller D. (2023). Multidrug-resistant *Pseudomonas aeruginosa* keratitis associated with artificial tear use. JAMA Ophthalmol..

[22] (2023). Centers for Disease Control and Prevention. Outbreak of Extensively Drug-Resistant Pseudomonas Aeruginosa Associated with Artificial Tears.

[23] Wang T., Jain S., Glidai Y., Dua P., Dempsey K.S., Shakin E., Chu D.S., Epstein M., Ha L.G. (2023). Extensively drug-resistant *Pseudomonas aeruginosa* panophthalmitis from contaminated artificial tears. IDCases.

[24] Kuo I.C. (2023). Extensively multi-drug-resistant *Pseudomonas aeruginosa* in artificial tears: Public health sleuthing success but challenges ahead. Am. J. Ophthalmol..

[25] Grossman M.K., Rankin D.A., Maloney M., Stanton R.A., Gable P., Stevens V.A., Ewing T., Saunders K., Kogut S., Nazarian E. (2024). Extensively drug-resistant *Pseudomonas aeruginosa* outbreak associated with artificial tears. Clin. Infect. Dis..

[26] Rezaei S., Steen D., Amin S. (2023). Successful treatment of an extensively drug-resistant pseudomonal ulcer associated with contaminated artificial tears. Am. J. Ophthalmol. Case Rep..

[27] Arun K., Georgoudis P. (2024). *Pseudomonas* Keratitis: From Diagnosis to Successful Deep Anterior Lamellar Keratoplasty. Cureus.

